# The kinetics and mechanism of H_2_O_2_ decomposition at the U_3_O_8_ surface in bicarbonate solution

**DOI:** 10.1039/d1ra05580a

**Published:** 2021-08-31

**Authors:** John McGrady, Yuta Kumagai, Masayuki Watanabe, Akira Kirishima, Daisuke Akiyama, Akira Kitamura, Shingo Kimuro

**Affiliations:** Nuclear Science and Engineering Center, Japan Atomic Energy Agency (JAEA) Tokai Ibaraki 319-1195 Japan mcgrady.john@jaea.go.jp kumagai.yuta@jaea.go.jp; Institute of Multidisciplinary Research for Advanced Materials, Tohoku University 1-1 Katahira, 2-chome, Aoba-ku Sendai 980-8577 Japan; Radionuclide Migration Research Group, Japanese Atomic Energy Agency (JAEA) Tokai Ibaraki 319-1195 Japan

## Abstract

In the event of nuclear waste canister failure in a deep geological repository, groundwater interaction with spent fuel will lead to dissolution of uranium (U) into the environment. The rate of U dissolution is affected by bicarbonate (HCO_3_^−^) concentrations in the groundwater, as well as H_2_O_2_ produced by water radiolysis. To understand the dissolution of U_3_O_8_ by H_2_O_2_ in bicarbonate solution (0.1–50 mM), dissolved U concentrations were measured upon H_2_O_2_ addition (300 μM) to U_3_O_8_/bicarbonate mixtures. As the H_2_O_2_ decomposition mechanism is integral to the dissolution of U_3_O_8_, the kinetics and mechanism of H_2_O_2_ decomposition at the U_3_O_8_ surface was investigated. The dissolution of U_3_O_8_ increased with bicarbonate concentration which was attributed to a change in the H_2_O_2_ decomposition mechanism from catalytic at low bicarbonate (≤5 mM HCO_3_^−^) to oxidative at high bicarbonate (≥10 mM HCO_3_^−^). Catalytic decomposition of H_2_O_2_ at low bicarbonate was attributed to the formation of an oxidised surface layer. Second-order rate constants for the catalytic and oxidative decomposition of H_2_O_2_ at the U_3_O_8_ surface were 4.24 × 10^−8^ m s^−1^ and 7.66 × 10^−9^ m s^−1^ respectively. A pathway to explain both the observed U_3_O_8_ dissolution behaviour and H_2_O_2_ decomposition as a function of bicarbonate concentration was proposed.

## Abstract 2

A transition from catalytic to oxidative H_2_O_2_ decomposition on U_3_O_8_ was observed with NaHCO_3_ with significant implications for uranium dissolution.

## Graphical abstract

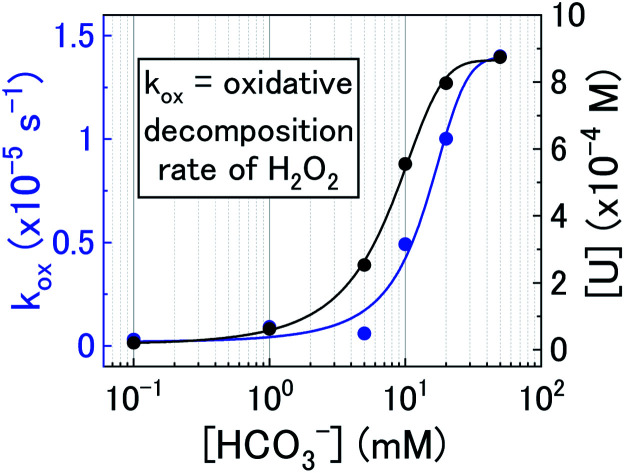

## Introduction

The current strategy for the disposal of spent nuclear fuel is in a deep geological repository according to the majority of the international community. The repositories provide a long-term storage solution, yet the release of radioactive species from spent nuclear fuel into the environment from the repository is projected to occur in the future upon failure of the repository barriers. Therefore, it is necessary to develop safety models for the repositories to predict their performance when failure occurs and nuclear material is exposed to the local environment. The main pathway for radionuclide release is predicted to be caused by the ingress of groundwater into the repository and interaction of the groundwater with the surface of the spent fuel. Understanding the reaction mechanisms between groundwater and spent fuel is integral to the development of safety models. Such interactions between the groundwater and spent fuel will lead to dissolution of the UO_2_ matrix which constitutes the majority of the spent fuel.^1^ The solubility of U in groundwater is governed by the form of U (U^(IV)^, U^(V)^ and U^(VI)^), with the hexavalent U^(VI)^ form being more soluble than U^(IV)^ and U^(V)^.^2–4^ Therefore, the presence of U^(VI)^ facilitates U dissolution into the groundwater upon canister failure.

Under the reducing, anoxic conditions typically found in groundwater at repository depths, the solubility of U^(IV)^ is very low,^5–7^ and so significant dissolution of the UO_2_ spent fuel may not be expected. However, radiation from the spent fuel will cause radiolysis of fuel adjacent water leading to the formation of a complex water chemistry involving radical, ionic and molecular species in the form of both reductants (e_aq_−, H^·^, H_2_) and oxidants (OH^·^, H_2_O_2_).^8^ This will significantly affect the local redox chemistry of the water and the oxidation state of U.

Of the oxidants generated by radiolysis, it has been shown that H_2_O_2_ is the dominant species in regards to U dissolution under deep geological repository conditions.^9,10^ The interaction of H_2_O_2_ with the UO_2_ surface has been thoroughly studied due to its importance for U dissolution, and it has been proposed that there are two competing pathways, both of which involve the decomposition of H_2_O_2_ at the UO_2_ surface.^11,12^ The first involves catalytic H_2_O_2_ decomposition forming H_2_O_2_ and O_2_ where the UO_2_ surface acts as a catalyst (ads = adsorbed):

1 (H_2_O_2_)_ads_ → 2(OH˙)_ads_

2
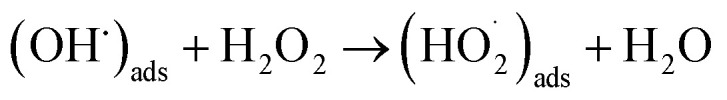
3
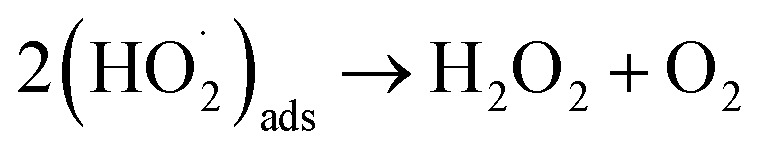


In this case, the H_2_O_2_ decomposition does not directly cause U dissolution. The second is an oxidative decomposition reaction where H_2_O_2_ oxidises U^(IV)^ to U^(V)^ (eqn (4)) and U^(V)^ to U^(VI)^ (eqn (5)) while itself being reduced to OH^−^ (eqn (6)) in a redox couple.

4 U^(IV)^O_2_ → U^(V)^O_2_+ + e^−^

5 U^(V)^O_2_ → U^(VI)^O_2_^2+^ + e^−^

6
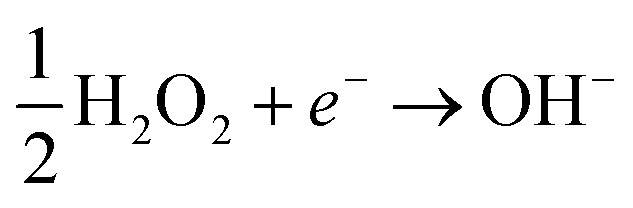


Typically, groundwater also contains bicarbonate (HCO_3_^−^) which has been shown to enhance the dissolution of U due to favourable complexation with U^(VI)^ and stabilisation of the dissolution products:^13–16^

7 U^IV^ + HCO_3_^−^ → U^V^(HCO_3_)_ads_ + e^−^

8 U^V^(HCO_3_)_ads_ + OH^−^ → U^VI^(CO_3_)_ads_ + e^−^ + H_2_O

9 U^VI^(CO_3_)_ads_ + HCO^−^_3_ → (U^VI^O_2_(CO_3_)_2_)^2−^ + H^+^

Therefore, the concentration of bicarbonate is believed to have a significant effect on U dissolution and the rate of H_2_O_2_ decomposition at the UO_2_ surface.

Due to the importance of developing models to predict U dissolution into groundwater, various studies have been undertaken with UO_2_ in simulated groundwater. A recent study by Kumagai *et al.*^17^ has shown that increasing the oxygen content from UO_2_ to UO_2.3_ increased U dissolution and reduced the rate of H_2_O_2_ decomposition at the oxide surface. As the dissolution of U is governed by the redox behaviour of the U atoms, it follows that the ratio of U^(IV)^, U^(V)^ and U^(VI)^ will have a significant impact on both U dissolution as well as the H_2_O_2_ decomposition pathway. Therefore, the form of uranium oxide that exists on the spent fuel oxide will have a large effect on the dissolution of U into the environment. Due to the radiolysis of spent fuel surface adjacent groundwater and the elevated temperatures from spent fuel decay, the formation of highly oxidised forms of U is expected *i.e.*, where *x* > 0.3 for UO_2+*x*_. However, there is still a lack of knowledge regarding the impact of higher oxidised forms of U on the mechanism of U dissolution and H_2_O_2_ decomposition.

To investigate this, we adopted U_3_O_8_ as an extreme case, which corresponds to UO_2.66_ containing two U^(V)^ atoms and one U^(VI)^ atom.^18–20^ U_3_O_8_ has been observed on used nuclear fuel both in wet^21^ and air^22,23^ environments, and can be used as a highly oxidised form of uranium oxide for an examination of the effects of U valence on U dissolution. As the complexation of bicarbonate with U^(VI)^ is thought to drive U dissolution by favourable complexation, the effect of U oxidation state on the dissolution of U in bicarbonate solution can be investigated by using U_3_O_8_. The H_2_O_2_ decomposition mechanism is dependent on U oxidation and so can also be investigated using U_3_O_8_ for comparison with UO_2_. The concentration of bicarbonate in groundwater is dependent on the location of the deep geological repository, and can range from ∼10^−4^ M (Tono, Japan),^24^ to ∼10^−3^ M (Daejeon, South Korea),^25,26^ to ∼10^−2^ M (Forsmark, Sweden)^27^ and so it is necessary to understand U dissolution and H_2_O_2_ decomposition at uranium oxide surfaces over a range of bicarbonate concentrations.

Therefore, in this work, U dissolution from U_3_O_8_ suspensions with H_2_O_2_ as a function of sodium bicarbonate (NaHCO_3_) has been investigated, and the mechanism of H_2_O_2_ decomposition at the U_3_O_8_ surface has been elucidated.

## Experimental

### Materials

Two samples of U_3_O_8_ powder were used in this study to investigate the reproducibility of the U dissolution tests. The first U_3_O_8_ powder (sample 1) was prepared by heating UO_2_ powder to 750 °C for 3 hours under a continuous flow of air. The second (sample 2) was prepared by dissolving U metal in 13 M HNO_3_ (Fujifilm Wako Pure Chemical, 60%) to form UO_2_(NO_3_)_2_·(H_2_O)_*n*_, which was then heated under identical conditions to give a 96% yield of U_3_O_8_. The formation of U_3_O_8_ was confirmed by XRD and the data was refined using the Rietveld method.^28^ The average crystallite size was measured using the Scherrer equation:^29^10
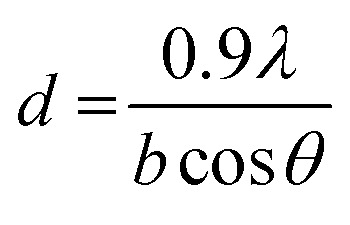
where *d* is the mean crystallite size, *λ* is the X-ray wavelength (1.5406 Å), *b* is the full width at half maximum value, and *θ* is the diffraction peak position. The crystallite sizes were calculated as 47 and 46 nm for sample 1 and 2 respectively. The orthorhombic lattice constants were also calculated from the diffractograms using Bragg's law^30^ for orthorhombic structures (1/*d*_*hkl*^2^_ = *h*^2^/*a*^2^ + *k*^2^/*b*^2^ + *l*^2^/*c*^2^) giving values of *a* = 6.72, *b* = 11.96 and *c* = 4.15 Å for sample 1 and *a* = 6.71, *b* = 11.95 and *c* = 4.14 Å for sample 2. The lattice constants were consistent with those for U_3_O_8_ (*a* = 6.72, *b* = 11.96 and *c* = 4.15 Å).^31^ This indicated that the structure of each U_3_O_8_ sample prepared *via* different methods was almost identical. Sample 1 was used for determination of the pseudo-first order rate constants for H_2_O_2_ decomposition to investigate the mechanism of decomposition. Sample 2 was used for determination of the second order rate constants for H_2_O_2_ decomposition at the U_3_O_8_ surface for comparison with UO_2_. This assignment was solely due to the amount of sample required to conduct each set of experiments – there was an insufficient amount of sample 1 for the second order experiments. The reproducibility of the dissolution tests with different U_3_O_8_ samples could then be analysed by comparison of H_2_O_2_ decomposition rates on each sample. The specific surface area of the powders was measured for calculation of the second order rate constants. Specific surface areas were measured by the Brunauer–Emmett–Teller method^32^ of adsorption/desorption using Kr gas with a Micromeritics Tristar II instrument. This method involves the adsorption of a monolayer of gas onto the surface of the powder at cryogenic temperatures, and the volume of adsorbed gas provides surface area information. Values of 1.20 m^2^ g^−1^ and 2.52 ± 0.2 m^2^ g^−1^ for sample 1 and sample 2 were obtained respectively. After the immersion tests, the U_3_O_8_ powder was dried under vacuum and analysed by Raman spectroscopy to investigate alterations to the U_3_O_8_ surface.

### Dissolution experiments

The effect of NaHCO_3_ (Alfa Aesar) on the dissolution of U_3_O_8_ powder by reaction with H_2_O_2_ (Fujifilm Wako Pure Chemical, 30%) was investigated by monitoring the U and H_2_O_2_ concentration as a function of reaction time. A suspension of U_3_O_8_ was prepared at concentrations of NaHCO_3_ between 0.1–50 mM (pH 8.2–9.7), and the suspensions were purged with Ar for approximately 18 hours to ensure removal of O_2_ to imitate the anoxic conditions of groundwater. Into the suspension, H_2_O_2_ was added to initiate the reaction. The concentration of H_2_O_2_ added was 300 μM which has been shown to be optimal to study oxidative dissolution on UO_2_.^17^ Ar purging was continued throughout the experiment. Experiments were conducted under atmospheric pressure at a temperature of 25 °C which was maintained with a coolant system. Samples of the suspension were taken at intervals over the course of the reaction. The samples were immediately filtered through a 0.45 μm filter to stop the reaction, and then analysed for H_2_O_2_ and U. For determination of the pseudo-first order rate constants, 50 mg of U_3_O_8_ (sample 1) was added to 50 ml bicarbonate solution, whilst for the second-order rate constant measurements, 50, 100, 150 and 200 mg of U_3_O_8_ (sample 2) were added to 70 ml bicarbonate solution. Error in the experimental methodology was estimated as <5% by conducting a set of dissolution experiments in triplicate and taking the standard deviation of the H_2_O_2_ pseudo-first order decay constants.

### Analytical techniques

The concentration of U was measured by ICP-OES using a PerkinElmer Avio-200 spectrometer. Calibration was conducted using U standards and measurements were done in triplicate. The standard deviations of the measurements were typically <1% of the measured values. The concentration of H_2_O_2_ was measured by the Ghormley triiodide method where the iodide ion (I^−^) reacts with H_2_O_2_ and is converted to triiodide (I_3_^−^) using ammonium heptamolybdate ((NH_4_)_6_Mo_7_O_24_) and an acidic buffer (KHC_8_H_4_O_4_).^33,34^ The concentration of H_2_O_2_ was then determined from the absorbance spectra of I_3_^−^ at 350 nm using a Shimadzu UV-3600 Plus UV-Vis-NIR spectrophotometer. Raman analysis of the oxide surface was conducted with a JASCO NRS-4500 Raman spectrometer. A 532 nm laser was introduced through a 20× objective lens, and 3 spectra of 10 seconds each were recorded and averaged for each sample.

## Results and discussion

### U dissolution

**Fig. 1:**
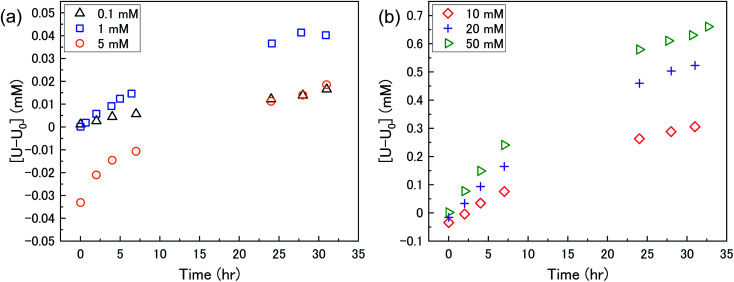
The dissolution of U as a function of time in a 50 mg suspension of U_3_O_8_ (sample 1) in (a) 0.1, 1 and 5 mM bicarbonate and (b) 10, 20 and 50 mM bicarbonate solution after addition of 300 μM H_2_O_2_.

The dissolution of U upon addition of H_2_O_2_ to U_3_O_8_ suspensions as a function of NaHCO_3_ was investigated by measuring dissolved U concentrations over the reaction time. Fig. 1 shows the dissolved uranium (U) minus the dissolved U concentration prior to H_2_O_2_ addition (U_0_). The dissolution of U changed over the experimental time and showed a clear effect of bicarbonate on the dissolution of U_3_O_8_. At 0.1 < [NaHCO_3_] < 5 mM, the dissolution was low. At > 5 mM the extent of dissolution significantly increased with bicarbonate. This is due to a change in the H_2_O_2_ decomposition mechanism as discussed later. The magnitude of U dissolution decreased with increasing bicarbonate concentration (*i.e.*, from 5 to 10 mM bicarbonate the increase in U dissolution was ∼0.3 mM, and from 20 to 50 mM was ∼0.1 mM) indicating a complex relationship.

**Fig. 2:**
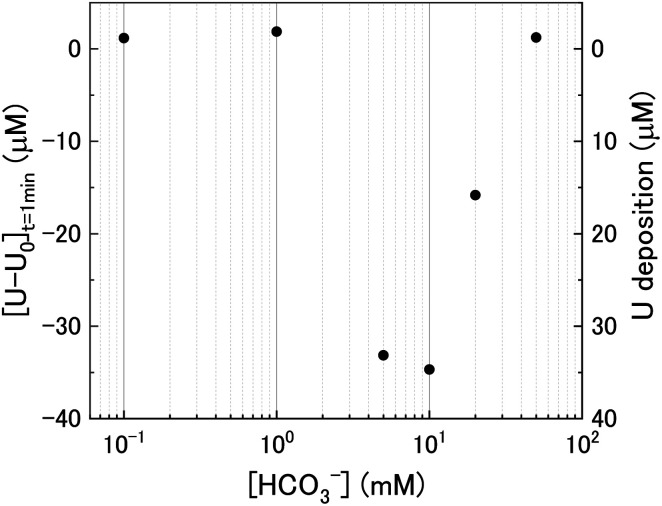
The initial change in U concentration one minute after H_2_O_2_ addition to the U_3_O_8_ suspension as a function of bicarbonate concentration.

At *t* = 1 min, the value of [U–U_0_] became negative at certain bicarbonate concentrations, and so the measured values of [U–U_0_]_*t*=1min_ were plotted as a function of bicarbonate concentration (Fig. 2). A decrease in the concentration of dissolved U can be seen in 5, 10 and 20 mM NaHCO_3_ solution, suggesting deposition from solution of U onto the U_3_O_8_ surface after the initial addition of H_2_O_2_ as highlighted by the second *y*-axis. As the extent of deposition increased with bicarbonate, it can be predicted that the deposits are uranium carbonates. Under the experimental conditions, the stable form of U in solution is UO_2_(CO_3_)_3_^4−^ and so the deposits may be UO_2_(CO_3_)_3_. Another possibility is the formation of uranyl peroxide (UO_2_(O_2_)), where the increase in deposition with bicarbonate is due to an increase in dissolved U with bicarbonate and, therefore, an increase in uranyl peroxide.

### Kinetics of H_2_O_2_ decomposition

**Fig. 3:**
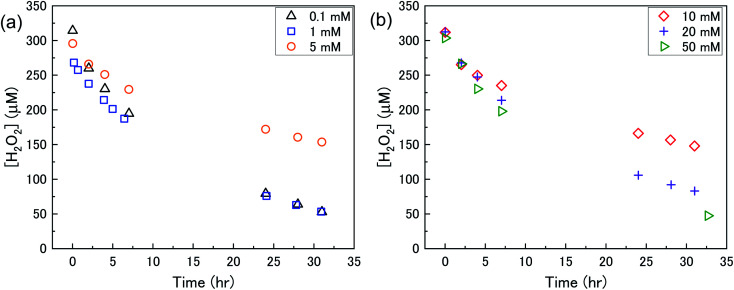
The concentration of H_2_O_2_ as a function of time in a 50 mg suspension of U_3_O_8_ (sample 1) in (a) 0.1, 1 and 5 mM bicarbonate and (b) 10, 20 and 50 mM bicarbonate solution after addition of 300 μM H_2_O_2_.

The dissolution of U from U_3_O_8_ was induced by the addition of H_2_O_2_ to the bicarbonate solution. Therefore, to understand the observed U dissolution behaviour from U_3_O_8_, the kinetics and mechanism of H_2_O_2_ decomposition at the U_3_O_8_ surface was studied. The kinetics of the reaction between H_2_O_2_ and U_3_O_8_ as a function of NaHCO_3_ were investigated by measuring the concentration of H_2_O_2_ over the reaction time. Fig. 3 shows the concentration of H_2_O_2_ after adding 300 μM to a 50 mg suspension of U_3_O_8_ at different bicarbonate concentrations as a function of time. The H_2_O_2_ concentration decreased quickly at low bicarbonate concentration (0.1 mM), but the decomposition slowed down as the bicarbonate concentration increased to 5 mM. Further increases in the bicarbonate concentration up to 50 mM caused the rate of H_2_O_2_ decomposition to gradually increase again, until the H_2_O_2_ concentration profile in 50 mM bicarbonate was similar to that in 0.1 mM bicarbonate. The results in Fig. 3 clearly show an effect of bicarbonate on H_2_O_2_ decomposition on U_3_O_8_.

**Fig. 4:**
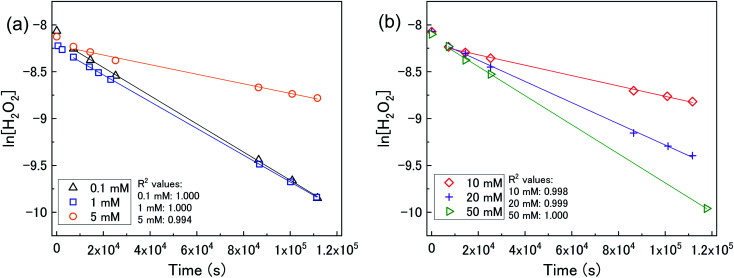
(a) A plot of ln[H_2_O_2_] *vs.* time as a function of bicarbonate concentration with 50 mg U_3_O_8_ (sample 1) in (a) 0.1, 1 and 5 mM bicarbonate and (b) 10, 20 and 50 mM bicarbonate solution showing pseudo-first order behaviour.

To investigate the mechanism of H_2_O_2_ decomposition at the U_3_O_8_ surface, the kinetics of decomposition was investigated. Previous studies on H_2_O_2_ decomposition at the surface of uranium oxides have shown that the reaction follows first order kinetics with respect to H_2_O_2_. As the U_3_O_8_ surface is in excess relative to H_2_O_2_, the reaction can be modelled as a pseudo-first order reaction. Therefore, the rate of H_2_O_2_ decomposition can be explained by,11
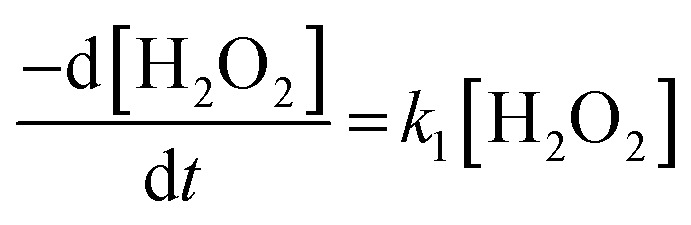
and the kinetics of H_2_O_2_ decomposition can be investigated by plotting the ln[H_2_O_2_] *vs.* time, where the gradient of the resulting straight-line plot gives the pseudo-first order rate constant, *k*, for the reaction (Fig. 4). The plots exhibited non-linear behaviour after the initial addition of H_2_O_2_ to the U_3_O_8_/bicarbonate mixtures indicating an initial reaction of H_2_O_2_ with the U_3_O_8_ surface. This initial fast decomposition of H_2_O_2_ is attributed to the formation of a surface layer and is further discussed later.

**Fig. 5:**
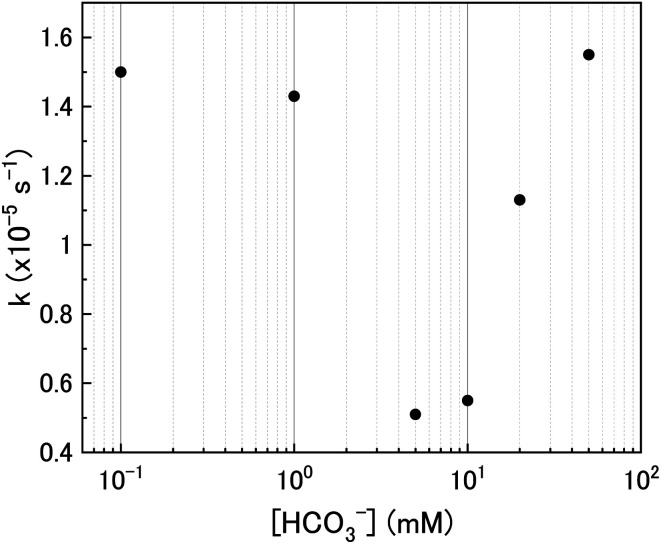
The pseudo-first order rate constants for H_2_O_2_ decomposition.

The calculated values of *k* from the linear region (from *t* = 2 hours to the experiment end) are plotted against bicarbonate concentration in Fig. 5 and the value of *k* was found to be in the range between 0.4 to 1.6 × 10^−5^ s^−1^. The decrease in the pseudo-first order rate constant coincided with U deposition from solution indicating that the secondary phases that deposit on the surface of the U_3_O_8_ may block the approach of H_2_O_2_ to the surface. As the plots in Fig. 4 show linear behaviour, this suggests that these deposits are stable over the experimental timescale.

### U dissolution with H_2_O_2_ decomposition

**Fig. 6:**
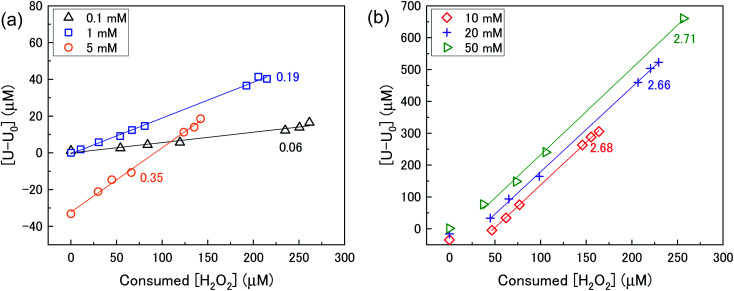
U dissolution from a 50 mg U_3_O_8_ (sample 1) suspension as a function of consumed H_2_O_2_ in (a) 0.1, 1 and 5 mM bicarbonate and (b) 10, 20 and 50 mM bicarbonate solution.

The mechanism of U dissolution *via* H_2_O_2_ decomposition can be investigated by analysing the extent of U dissolution as a function of H_2_O_2_ decomposition. To illustrate this, Fig. 6 shows a plot of the amount of dissolved U against the amount of consumed H_2_O_2_ for each bicarbonate concentration. The U dissolution per H_2_O_2_ decomposition shows linear behaviour. If we consider the oxidative pathway for H_2_O_2_ decomposition at the U_3_O_8_ surface, U^(V)^ is oxidised to U^(VI)^ leading to decomposition of the U_3_O_8_ unit since the net charge in the lattice is no longer neutral. Therefore, each H_2_O_2_ decomposition event *via* oxidative decomposition will lead to a U_3_O_8_ dissolution event (U_3_O_8_ + H_2_O_2_ → 3UO_2_^2+^_(aq)_), and a gradient of 3 may be expected from the plots in Fig. 6. If we consider only catalytic decomposition of H_2_O_2_, no U dissolution would occur giving an ideal gradient of 0. Therefore, the measured gradients provide a ratio of oxidative to catalytic H_2_O_2_ decomposition at each bicarbonate concentration, assuming these pathways are the only pathways for H_2_O_2_ decomposition (*i.e.* for 50 mM bicarbonate the ratio of oxidative dissolution is 
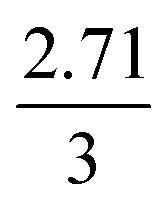
 and catalytic decomposition is 
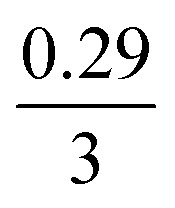
). The dissolution of U from U_3_O_8_ in 10 mM bicarbonate gave a gradient of 2.68. By comparison with the gradients measured from the dissolution of U from UO_2_ (∼0.4) and UO_2.3_ (∼1) with H_2_O_2_ addition in 10 mM bicarbonate,^17^ this shows that the oxidative dissolution of U increases with increased oxidation state of the uranium oxide. As the complexation of U^(VI)^ with bicarbonate drives the dissolution of U from the surface, it follows that the two-step oxidation of U^(IV)^ → U^(V)^ → U^(VI)^ for UO_2_ would result in less dissolution of U than the one-step oxidation for U^(V)^ → U^(VI)^ in U_3_O_8,_ and for intermediate UO_2+*x*_ stoichiometries the dissolution rate would increase with increasing values of *x*. Another point of consideration regarding U dissolution is the crystal structure of UO_2_ (cubic fluorite) and U_3_O_8_ (orthorhombic). As the crystal structures are different, the number of surface sites for H_2_O_2_ decomposition will impact U dissolution. The surface site densities for UO_2_ and U_3_O_8_ have been reported as between 126 (ref. 35) to 165 (ref. 36) sites per nm^2^ for UO_2_ and 48 (ref. 36) sites per nm^2^ for U_3_O_8_. As the surface sites are ∼3 times lower for U_3_O_8_, the observed increase in U dissolution from U_3_O_8_ relative to UO_2_ is more pronounced than the measured dissolved U concentrations suggest.

**Fig. 7:**
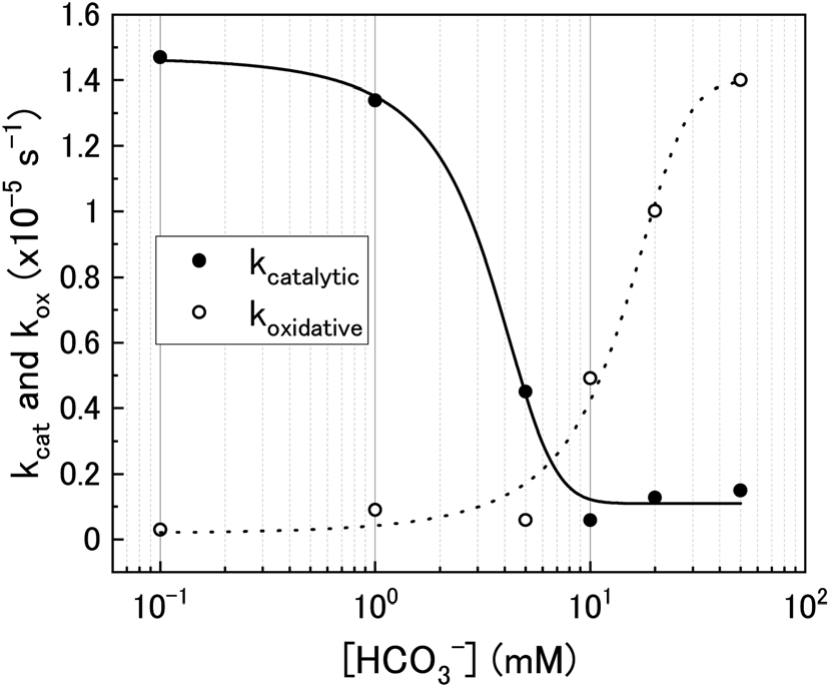
The catalytic (*k*_cat_) and oxidative (*k*_ox_) pseudo-first order rate constants for H_2_O_2_ decomposition on U_3_O_8_ as a function of bicarbonate concentration.

Using the ratios taken from the gradients, the contributions of catalytic (*k*_cat_) and oxidative (*k*_ox_) decomposition to the measured pseudo-first order rate constant can be found and are plotted in Fig. 7 as a function of bicarbonate.

**Fig. 8:**
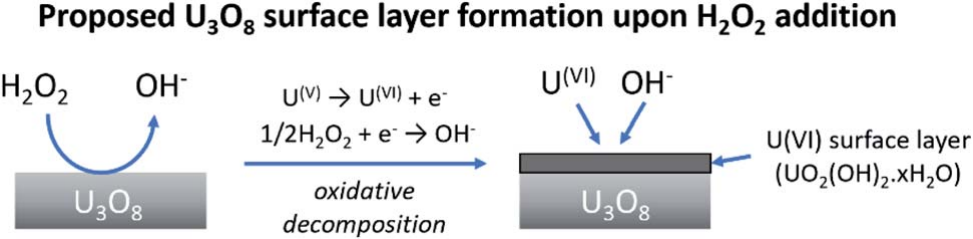
The formation of a protective surface layer on the surface of U_3_O_8_ due to oxidative decomposition of H_2_O_2_.

At low bicarbonate concentrations, the main pathway for H_2_O_2_ decomposition is the catalytic decomposition mechanism as there is little U dissolution associated with H_2_O_2_ decomposition. As *k*_ox_ is low, this indicates that the U_3_O_8_ is protected from H_2_O_2_ by a surface layer. It is postulated that upon addition of H_2_O_2_ to the bicarbonate solution, oxidative dissolution proceeds on the bare U_3_O_8_ surface (Fig. 8). As this involves the oxidation of U^(V)^ to U^(VI)^, it is likely that U^(VI)^ forms a surface layer on the U_3_O_8_ which protects against further oxidative dissolution as U^(VI)^ is already fully oxidised, and due to the low concentration of bicarbonate the surface layer is stable.

**Fig. 9:**
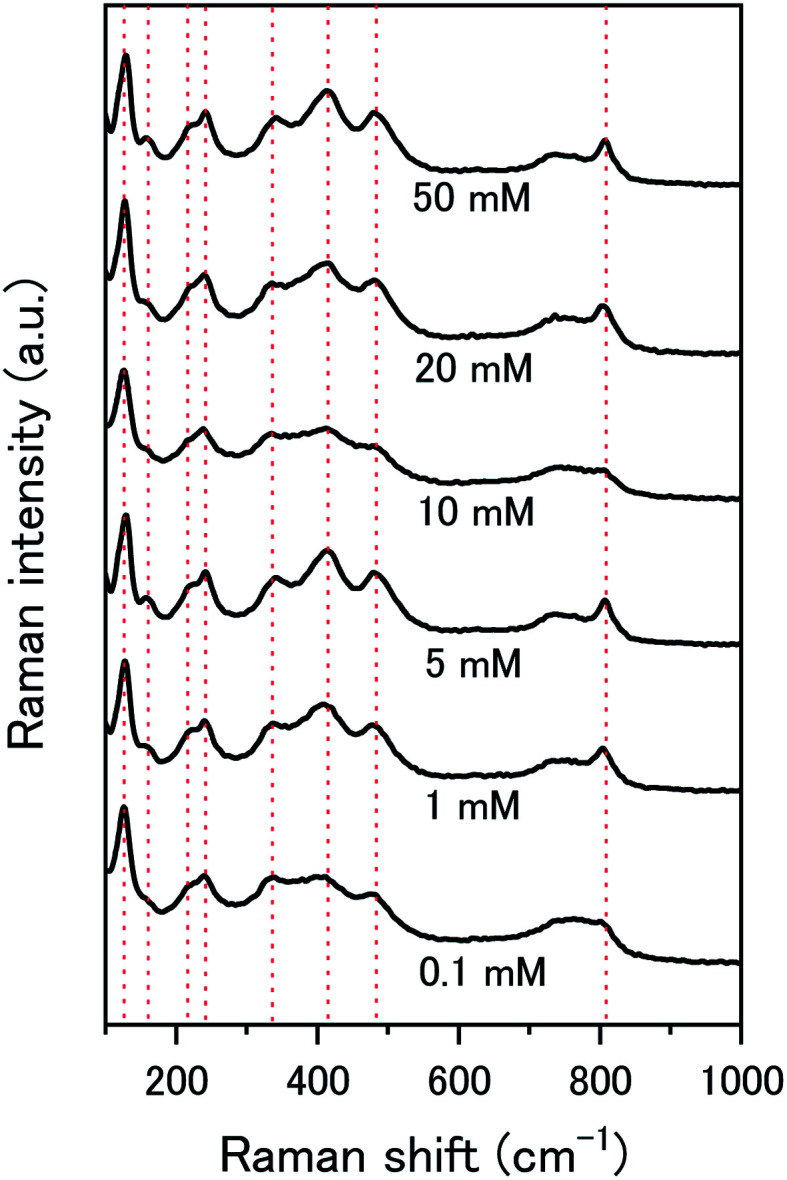
Raman spectra of U_3_O_8_ (sample 1) after the dissolution tests for different concentrations of NaHCO_3_.

The composition of the surface layer is thought to be in the hydroxide form (UO_2_(OH)_2_·*x*H_2_O) due to the formation of hydroxide from the oxidative decomposition of H_2_O_2_. Raman analysis of the U_3_O_8_ surface after removal from solution and vacuum drying showed spectra representative of U_3_O_8_ only (Fig. 9). Peaks relating to U_3_O_8_ were observed including the U–O A1_g_ stretching modes at 335 and 410 cm^−1^, and the U–O E_g_ stretching mode at 475 cm^−1^.^37^ As U_3_O_8_ was the only phase observed, any surface layer that formed had been removed prior to Raman analysis. If the surface layer is in the hydroxide form, it is expected to decompose upon drying which would explain the observed results. Further studies are required to elucidate the composition of the surface.

As the bicarbonate concentration increases from 0.1 to 5 mM, the rate of catalytic H_2_O_2_ decomposition decreases. This is caused by an increase in deposition from solution as seen in Fig. 2. As the pseudo-first order rate constant decreases up to 5 mM, it can be said that the deposits do not catalyse H_2_O_2_ decomposition to the extent that U_3_O_8_ does.

Increasing the bicarbonate concentration > 5 mM changes the main H_2_O_2_ decomposition mechanism pathway from catalytic to oxidative. At NaHCO_3_ concentrations of 10 mM and above, at least 90% of the H_2_O_2_ decomposed *via* oxidation of U^(V)^ to U^(VI)^. This is due to increased dissolution of the U^(VI)^ surface layer and exposure of the U_3_O_8_ surface beneath leading an increase in *k*_ox_. As the value of *k* increases with bicarbonate, this suggests that the dissolution step is rate determining rather than the redox reaction. Therefore, dissolution experiments in solutions of higher bicarbonate concentrations are required to elucidate the true value of *k* for oxidative dissolution in this system. Interestingly, a study by Nilsson *et al.* on UO_2_ dissolution in 10 mM NaHCO_3_ with H_2_O_2_ addition using pellets showed that ∼14% of H_2_O_2_ decomposition events occurred *via* oxidative dissolution while the value was even lower (∼2%) on SIMFUEL.^38^ This suggests that *k*_cat_ is high in the case of the pellets indicating that the surface oxide that forms on the pellets is more protective than on the powders. The large discrepancy between the H_2_O_2_ decomposition behaviour between UO_2_ pellets and U_3_O_8_ powder (and UO_2_ powder) is a point that requires investigation.

**Fig. 10:**
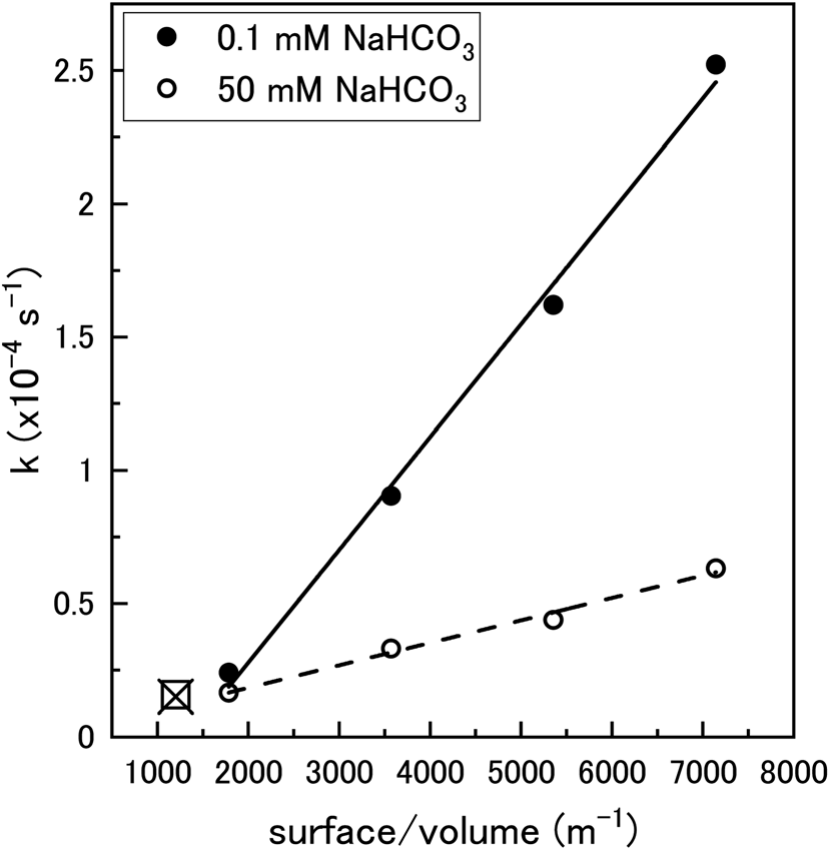
The pseudo-first order rate constant, *k*, plotted against the U_3_O_8_ (sample 2) surface area to solution volume ratio (m^−1^) for 0.1 mM and 50 mM HCO_3_^−^ solutions (70 ml solution). The data for 0.1 mM (□) and 50 mM (x) calculated using U_3_O_8_ sample 1 shown in Fig. 5 is included to show reproducibility (50 mg U_3_O_8_ in 50 ml solution).

To clarify the dependence of the catalytic and oxidative mechanisms on U_3_O_8_, the second order rate constants for H_2_O_2_ decomposition were obtained for 0.1 mM and 50 mM solutions with U_3_O_8_ (sample 2). The second order rate equation,12
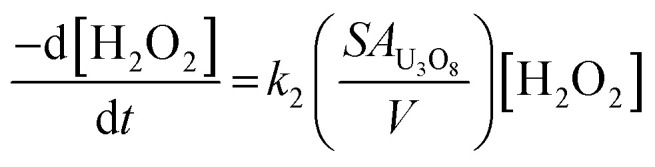
can be used to obtain the second order rate constant by plotting the pseudo-first order rate constant against the U_3_O_8_ surface area to total solution volume ratio (Fig. 10). The second order rate constant in 0.1 mM bicarbonate was 4.24 × 10^−8^ m s^−1^. At this concentration, the decomposition was shown to be almost completely catalytic, and so this can be attributed to the catalytic decomposition reaction pathway shown in eqn (1)–(3). At 50 mM, the value of the measured second order rate constant was 8.44 × 10^−9^ m s^−1^, and as the ratio of oxidative decomposition was ∼90%, we can estimate the oxidative decomposition rate constant to be 7.60 × 10^−9^ m s^−1^ for the pathway shown in eqn (5) and (6). These values are within the range described in the literature for catalytic decomposition (3.6 × 10^−8^ to 5 × 10^−11^ m s^−1^) and oxidative decomposition (1.4 × 10^−7^ to 2.0 × 10^−10^ m s^−1^) of H_2_O_2_ at the UO_2_ surface.^39^ The pseudo-first order rate constant measurement for 0.1 mM and 50 mM bicarbonate solutions using U_3_O_8_ sample 1 (shown in Fig. 5) are included in Fig. 10 showing the reproducibility of the data using different U_3_O_8_ powders.

### Proposed pathway for U_3_O_8_ dissolution by H_2_O_2_ in NaHCO_3_ solution

**Fig. 11:**
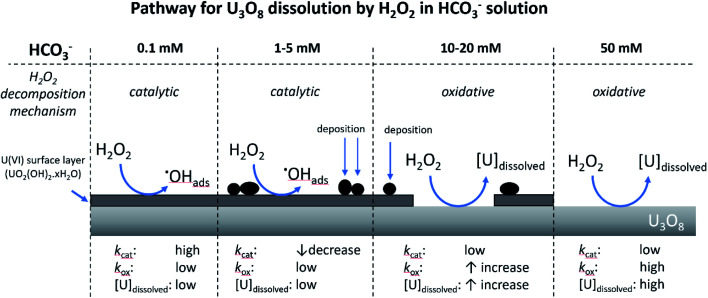
The proposed pathway for U_3_O_8_ dissolution upon H_2_O_2_ addition as a function of bicarbonate concentration.

From the experimental results, a proposed pathway to explain the observed behaviour of U_3_O_8_ in bicarbonate solution with H_2_O_2_ is summarized, and a schematic is provided in Fig. 11. At low bicarbonate concentrations upon H_2_O_2_ addition, oxidative decomposition of H_2_O_2_ occurs at the exposed U_3_O_8_ surface forming a surface layer comprised of U^(VI)^ that provides protection against further oxidative dissolution. The decomposition of H_2_O_2_ proceeds *via* catalytic decomposition, and so the rate of U dissolution is low. The surface layer protects the U_3_O_8_ in bicarbonate concentrations up to 5 mM, and the H_2_O_2_ decomposition mechanism remains catalytic and U dissolution remains low. At 10 mM bicarbonate, the concentration of bicarbonate is sufficient to induce dissolution of the surface layer, and the surface layer does not fully protect the U_3_O_8_ which is exposed leading to oxidative decomposition of H_2_O_2_ and an increase in U dissolution. At higher bicarbonate concentrations, the surface layer is further dissolved, and oxidative decomposition of H_2_O_2_ and dissolution of U proceeds at higher rates.

## Conclusions

Based on the presented results, the effect of bicarbonate on U dissolution from U_3_O_8_ with H_2_O_2_ addition can be split into 3 sections:

(1) [NaHCO_3_] < 5 mM: H_2_O_2_ decomposition occurs *via* catalytic decomposition at the U_3_O_8_ surface, and the dissolution of U into solution is low.

(2) 5 mM < [NaHCO_3_] < 20 mM: secondary phases deposit onto the surface of the U_3_O_8_ upon H_2_O_2_ addition. The mechanism of decomposition changes from catalytic to oxidative, causing dissolution of U.

(3) [NaHCO_3_] > 20 mM: the decomposition mechanism of H_2_O_2_ is >90% oxidative, leading to significant dissolution of U.

The concentration of bicarbonate and form of uranium oxide has a large influence on U dissolution and H_2_O_2_ decomposition. Significant dissolution of U from U_3_O_8_ was observed at bicarbonate concentrations > 5 mM, and the extent of U dissolution was found to be larger on U_3_O_8_ than for UO_2_ which was attributed to the one-step oxidation of U^(V)^ to U^(VI)^ for U_3_O_8_ compared to the two-step oxidation for UO_2_ from U^(IV)^ to U^(V)^ to U^(VI)^. The rate of H_2_O_2_ decomposition on U_3_O_8_ was comparable to literature data for UO_2_. However, the mechanism of H_2_O_2_ decomposition on U_3_O_8_ showed a strong dependence on the concentration of bicarbonate in solution with catalytic preferred at low bicarbonate and oxidative at high bicarbonate. The increase in catalytic activity at low bicarbonate was attributed to oxidation of the U_3_O_8_ surface and formation of a surface oxide.

Predicting the dissolution behaviour of spent fuel in the far future upon deep geological repository failure is a challenging task that requires significant experimental data for the development of accurate predictive models. In this work, elucidation of the mechanism of H_2_O_2_ decomposition on U_3_O_8_ and its effect on U dissolution was achieved, along with H_2_O_2_ decay constants as a function of simulated groundwater bicarbonate concentration. In groundwater containing high bicarbonate concentrations, significant dissolution of U from U_3_O_8_ is expected. This provides contributions to the development of such models for safety assessment of deep geological repositories. By demonstrating that the form of U will play a major role in the rate of U dissolution into the environment, the need for further studies regarding the effect of spent fuel composition on radionuclide dissolution into groundwater has been highlighted.

## Conflicts of interest

There are no conflicts to declare.

## Supplementary Material
